# The casualty chain inventory: a new scale for measuring peritraumatic responses: a cross-sectional study

**DOI:** 10.1186/1471-227X-11-6

**Published:** 2011-05-18

**Authors:** Laila Skogstad, Erlend Hem, Leiv Sandvik, Øivind Ekeberg

**Affiliations:** 1 Department of Research and Development, Division of Critical Care, Oslo University Hospital, Ulleval, Norway; 2 Department of Behavioural Sciences in Medicine, Faculty of Medicine, University of Oslo, Oslo, Norway; 3 Section of Epidemiology and Biostatistics, Oslo University Hospital, Ulleval, Norway; 4 Department of Acute Medicine, Oslo University Hospital, Ulleval, Norway

## Abstract

**Background:**

Peritraumatic psychological- and sensory impressions in victims of civilian accidents are only partly understood. This study scrutinizes the level and duration of perceived psychological threat at *scene of injury *as well as *in hospital *(the casualty chain) measured by the Casualty Chain Inventory (CCI). The purpose of the study was to assess and validate the CCI, and to examine the correlations between the new instrument and stress responses measured by the Impact of Event Scale (IES) and the Post-traumatic Stress Scale-10 (PTSS-10)

**Methods:**

Three hundred and fifteen injured, conscious, hospitalised patients were assessed with a self-report questionnaire. The CCI consists of eight items including sensory impressions and well-known psychological responses to trauma.

**Results:**

The internal consistency of the CCI was solid (Cronbach's alpha: .83-.85). A factor analysis revealed two components, "perception" and "dissociation". The instrument correlates significantly with the Impact of Event Scale (r = 0.47 - 0.54) and the Posttraumatic Stress Scale-10 (r = 0.32 - 0.50). The explained variance is high both at the scene of injury (61%) and in the hospital (65%). Dissociation and perception either used as a two-factor solution or as a sum score measured in the hospital, gave the strongest prediction for later psychological distress.

**Conclusions:**

The CCI appears to be a useful screening instrument for, at an early state, identifying patients hospitalized after a physical incident at risk for subsequent psychological distress.

## Background

Patients who are hospitalized because of physical traumas may perceive varying degrees of psychological threat during the incident. A physical accident can be viewed as a chain of connected moments--the moment one realize that something bad is about to happen, when it happens, waiting for help, transportation to, and treatment in hospital. This process may be defined as *the casualty chain*. The authors of two meta-analyses recommended further research on peritraumatic responses [1,2]. Ôzer et al. concluded that peritraumatic psychological processes might be the strongest predictors of posttraumatic stress disorder (PTSD). In order to get a diagnosis of posttraumatic stress disorder (PTSD), a high level of posttraumatic stress symptoms (PTS) must be present together with other diagnostic criteria.

Most instruments are developed to measure posttraumatic stress, such as the Impact of Event Scale [3], the Trauma Screening Questionnaire [4], and the Post-traumatic Stress Scale-10 [5]. However, only few instruments measure peritraumatic experiences including two that are commonly used, the Peritraumatic Dissociative Experiences Questionnaire [6] and the Peritraumatic Distress Inventory [7]. Peritraumatic dissociation has been assessed as one of the substantial predictors of post traumatic stress disorder (PTSD) [8] but may in fact be a confounding variable [9-11], and peritraumatic responses other than dissociation and peritraumatic distress connected to criterion A2 [7] might have an impact. Intrusive memories may often consist of sensory impressions, especially visual, of the moments preceding the traumatic event [12] and trauma-focused cognitive therapy often address the sensory influences on psychological responses during the event. How a person perceive and respond during the incident may also be associated with posttraumatic stress. It may also be possible to view pain as a sensory impression when physically injured since the tactile sense may be affected by the damage to the skin. Even though pain is a significant risk factor in physical trauma, the level of pain is also influenced by psychological factors [13]like the level of fear or autonomy. In addition, intense psychological despair (anxiety or depression) may also be painful even in the absence of physical injury. Pain may also increase the feeling of fear, and fear may increase the perception of pain. Therefore, the Casualty Chain Inventory (CCI) was developed, which includes sensory impressions and well-known psychological responses experienced both at *the scene of injury *and *in hospital *in order to get a measure of the duration of the responses. By measuring perceived threat at two time points, it is possible to see whether those with a high level of perceived threat both *at the scene of injury *and *in hospital *are at greater risk than those who are equally afraid in one of these situations, i.e. a measure of the duration of experienced threat.

The main contributions of the new instrument are the ability to measure peritraumatic sensory perception and the ability to compare the relative contribution of the sensory perception and items representing fear, dissociation and lack of autonomy (feeling stuck) for subsequent posttraumatic symptoms.

The purpose of the present study was to assess and validate the CCI, and to examine the correlations between the CCI and stress responses measured by the Impact of Event Scale (IES) and the Post-traumatic Stress Scale-10 (PTSS-10).

## Methods

### Procedures and Design

The CCI was tested on physically injured, conscious patients admitted to the emergency room at Oslo University Hospital Ulleval. The cross-sectional data was collected after discharge. Participants received a consent form, self-report questionnaire, and stamped return envelope approximately one - two weeks after discharge, with a reminder after one month. For the CCI they were asked to recall impressions and psychological responses experienced at *the scene of the injury *and *in the hospital*. Demographic characteristics were obtained, and for the IES and PTSS-10 the patients were asked to answer referring to the last seven days. Ulleval Trauma Registry provided data on the physical traumas.

The respondents had to be between 18-65 years. Measuring perceptions at scene of injury and in hospital required conscious patients and a Glasgow Coma Score (GCS) [14] equal to or above 11 was an inclusion criterion. A score of 3 indicates no response, and 15 reflect a normal level of consciousness. The participants returned the baseline questionnaires before randomization to an intervention (the intervention data will be reported in a subsequent paper), therefore living more than 60 kilometers from the hospital was an exclusion criterion. Patients were also excluded if they were unable to speak or read Norwegian or had unknown address, had self-inflicted injuries, had serious psychiatric and/or substance abuse problems (psychotic and/or in need of acute psychiatric treatment), and/or were involved in criminal acts.

### Measures

#### The Casualty Chain Inventory

A study group that consisted of a liaison psychiatrist, one medical doctor (who treats patients with physical trauma) and a trauma nurse developed the CCI. In this paper perceived threat reflects how the patient experiences impressions and responses during the incident, and the level of perceived threat both at *the scene of the injury *and *in the hospital*. The CCI contains eight items: fear, pain, visual, auditory and olfactory impressions, feeling emotionally stuck (i.e. lack of autonomy), feeling as if the situation was unreal and emotional numbness. This reflects a combination of psychological responses and sensory impressions, all based on how one may perceive a physical incident. The last two items were taken from the IES and represent responses related to dissociation more than avoidance. Response alternatives ranged from 1 (not at all) to 5 (to a very high degree). The combination of items was collected to study if responses other than dissociation and fear have an impact on posttraumatic stress.

#### Impact of Event Scale (IES)

The Norwegian translation of the **IES **has six response alternatives, from 0 (never) to 5 (a high degree), with scores ranging from 0 to 75. Seven items measure intrusion, and eight measures avoidance. It has been used in previous Norwegian studies on a similar sample of patients [3,15-17].

#### Post-Traumatic Stress Scale-10 (PTSS-10)

**PTSS-10 **is a 10-item scale, which measures posttraumatic stress symptoms including hyperarousal on a Likert scale where 1 represents "never/seldom" and 7 represents "very often", with scores ranging from 10 to 70 [5,18,19].

### Data Analysis

Statistical analyses were performed with SPSS, version 15.0 and included Spearman's rho, Student's *t *tests, and a principal components analysis with orthogonal (Varimax) rotation. A two-by-two-by-two analysis of covariance was performed to study demographic data and the CCI. The internal consistency of the scale was examined with Cronbach's alpha and correlation between "Item-score- Total-score". If no more than one item had a missing value, the mode of the other items replaced the missing value. One missing value per subscale was permitted. Data are presented as means, medians, 95% Confidence Intervals (CI) and SDs. Five percent of CCI data for *at the scene of the injury *questions and 3% of the data for *in the hospital *questions was missing.

### Ethics

The Norwegian Data Inspectorate and The Regional Ethics Committee approved the study.

## Results

### Participants

Three hundred and fifteen of 541 eligible physically injured patients (58%) admitted to the Emergency Room (ER) participated. The mean age was 38.7 years (range = 18-65), and 65% were men. Approximately half of the participants were married and 39% had custody of children. Over 85% were occupied in work or studies. The mean value of Glasgow Coma Scale was 14.9 (95% CI 14.8 - 14.9). Sixty-six percent were motor vehicle accidents, 17% falls, 8% assaults and 9% other incidents. The mean time between the trauma and the assessment was 29.4 days (95% CI 26.8 - 32.0) and median time was 22.0 days (range = 1-131). One quarter responded within two weeks and 62% had answered the questionnaire between two and eight weeks. The mean length of stay in hospital (LOS) was 4.0 days (95% CI 3.4 - 4.7) and median LOS was 2.0 days (range 0 - 52).

### Validation of the Casualty Chain Inventory

The highest mean scores occurred for the variables *pain *and *feeling emotionally stuck*, both at *the scene of the injury *and *in the hospital *(Table 1). A paired sample *t *test showed a statistically significant decrease in scores of all items from *the scene of the injury *(mean 2.6, SD = .90) to *in the hospital *(2.3, SD = .84), t(298) = 6.67, *p *< .001 (two-tailed). Correlations between Item-score and Total-score were high both *in the hospital *and at *the scene of the injury *(range .45-.75). Correlation between both measurement time points was .69 and Cronbach's alpha was strong at both measurement points. We entered the mean scores for the CCI at place of injury and in hospital (data not shown), and found that the CCI value in hospital was a stronger predictor for PTS symptoms.

**Table 1:** Items on the Casualty Chain Inventory at *the scene of the injury *and in the hospital * (means, correlations, and internal consistency)

	**At scene of injury**				**In the hospital**			
	**Item**	**Score**		**Correlation Between** **Item Score and** **Total Score**	**Item**	**Score**		**Correlation Between** **Item Score and** **Total Score**
**Item description;**								
**Rate the extent to which you**	**n**	**Mean**	**SD**		**n**	**Mean**	**SD**	
		
1. were afraid	300	2.9	1.4	.62	307	2.3	1.2	.62
2. had severe pain	302	3.5	1.3	.47	306	3.2	1.2	.48
3. had severe visual impressions	301	2.3	1.2	.67	304	2.1	1.7	.71
4. had severe auditory impressions	300	2.1	1.2	.66	303	2.0	1.1	.75
5. had severe olfactory impressions	298	1.6	.9	.52	305	1.7	.9	.63
6. felt emotionally stuck	298	3.0	1.5	.56	305	2.8	1.4	.59
7. felt as if the situation was unreal	302	2.7	2.1	.45	306	2.4	1.3	.50
8. felt emotional numbness	303	2.6	1.4	.49	304	2.2	1.3	.59
								
Correlation between scene of injury and in hospital = .69								
Cronbach's alpha				.83				.85

n = 315. *Scores range from 1 to 5 (not at all to a very high extent).

### Factor Analysis

Table 2 shows a two factor structure: (1) perception, with six variables (fear, pain, visual-, auditory and olfactory impressions, feeling emotionally stuck); and (2) dissociation, with two variables (feeling as if the situation was unreal, emotionally numbness).

**Table 2:** Factor analysis of the Casualty Chain Inventory

	*At scene of injury*		*In the hospital*	
		
Items	Perception	Dissociation	Perception	Dissociation
				
Fear	0.79		0.70	
Auditory impressions	0.77		0.84	
Pain	0.69		0.62	
Emotionally "stuck"	0.69		0.60	
Visual impressions	0.71		0.83	
Olfactory impressions	0.60		0.73	
Feeling of unreality		0.88		0.91
Emotional numbness		0.83		0.86
Cronbach's alpha	.83	.74	.84	.80

Rotated component with Varimax of two-factor solution

Factor 1 (perception) had eigenvalue of 3.73 and explained 47% of the variance. Factor 2 (dissociation) had eigenvalue of 1.14 and explained additionally 14% of the total variance at *the scene of the injury. In hospital*, the eigenvalues were 4.06 and 1.10, explaining 51% and additionally 14% of the variance, respectively. Cattell's scree plot revealed a break after the second component, confirming the two-factor structure. For dissociation the mean value was 2.6 (95% CI 2.4 - 2.8) at *scene of injury *and 2.3 (95% CI 2.1 - 2.5) *in hospital*. Mean score for perception was 2.5 (95% CI 2.4 - 2.7) at *scene of injury *and 2.3 (95% CI 2.2 - 2.4) *in hospital*. Using a sum score for dissociation and perception, the mean was 2.5 (95% CI2.4 - 2.7) at *scene of injury *and 2.3 (95% CI 2.2 - 2.4) *in hospital*. The Kaiser-Meyer-Olkin value was .81 at *the scene of injury *and .83 *in the hospital*. Bartlett's test of sphericity reached statistical significance, supporting the factors. Cronbach's alpha for the perception factor was .83 at *the scene of the injury *and .84 *in the hospital*. The corresponding figures for the dissociation factor were .74 and .80, respectively. To address a possible problem using two items from IES in the CCI a sum score without the two items were made. The CCI was compared with the IES both with and without the dissociation items, showing no significantly different correlations. The Cronbach's alpha for perception without the items was quite similar to the sum score with the items (*scene of injury *.49 vs. .47, and *in hospital *.54 for both sum scores). The corresponding Cronbach's alpha for dissociation was .43 at *scene of injury *and .48 *in hospital *without the items and .47 at scene of injury and .52 *in hospital *with the items.

### Relationship between the CCI and stress responses

The Cronbach's alpha for IES was .94 (total score), .93 (intrusion) and .90 (avoidance). For the PTSS-10 the Cronbach's alpha was .92. The correlations between the CCI and measures of posttraumatic stress (Table 3) were all moderately high (*r *= .32-.54) and highly significant (*p *< .001).

**Table 3:** Correlations between the CCI subscales and stress responses

	**At scene of injury**		**In the hospital**	
		
	**Perception**	**Dissociation**	**Perception**	**Dissociation**
	**n = 284**	**n = 301**	**n = 300**	**n = 304**
			
IES				
Total	.47**	.47**	.54**	.52**
Intrusion	.49**	.39**	.52**	.42**
Avoidance	.40**	.48**	.49**	.54**
PTSS-10	.40**	.32**	.50**	.41**

** Correlation is significant at the .01 level (two-tailed)

## Discussion

The CCI showed a two-factor structure of perception of the incident (6 items) and dissociation (2 items) with good internal consistency both at *the scene of the injury *and *in the hospital*. Even though there is a two-factor structure, the high Cronbach's alpha for all eight items indicates that a sum score of the CCI also can be used. The main contribution of the new instrument is the assessment of peritraumatic sensory perception. The factor analysis showed that dissociation was a separate factor, correlating with the perception factor (*at scene of injury *.42 and *in hospital *.46). ln this study, the level of fear however, loaded on the same factor as the perception items. It remains to be seen whether perception will be a stronger predictor for later PTS. The explained variance is high both at the scene of injury (61%) and in the hospital (65%). This means that about two-thirds of perceived threat is explained by the CCI and the instrument appears to be valid. The internal consistency of the CCI was somewhat lower compared to the IES and PTSS-10. Examining the association between the CCI score and the scores on IES and PTSS-10 was important to see whether the instruments assessed different phenomena. Since the correlation was < .7 the new instrument (CCI) can give further contribution when predicting posttraumatic stress symptoms.

Among the sensory impression scores, pain had the highest mean score. Fear decreased the most from *the scene of the injury *to *the hospital*. Even though mean scores for the sensory impressions were low, both the total scale and the factor perceptions showed strong internal consistency. This indicates that, in addition to dissociation, sensory impressions (together with fear and feeling emotionally stuck) are important to screen for when identifying who is at risk of developing clinically significant posttraumatic stress. Pain may both be a sensory perception as well as a psychological reaction and related to fear; this may explain the strong impact in this findings. It is possible that an activated stress response makes a person more vulnerable to sensory impressions. Therefore, the problem may involve a combination of high levels of sensory impact and heightened vulnerability to sensory impressions. In clinical work a screening of sensory impressions may also indicate which specific sensory channel (e.g. visual or auditory) to address in treatment of posttraumatic stress. Perception may have changed over time during the hospital stay and even after discharge. We have no data to address this issue, which may be a focus of future studies.

The CCI has some conceptually common items with other measures (feeling as if the situation was unreal, emotional numbness and fear) and some related phenomena (feeling emotionally stuck and sensory impressions). It would be pretentious to address this as convergent validity, but the commonalities are great enough to warrant studying the relationship between the CCI, the IES and the PTSS-10. The correlations with these stress measures were significant, indicating that the CCI can be used as a predictor for posttraumatic stress after injuries. It might have been interesting to assess the convergent validity with other measures of peritraumatic responses like the Peritraumatic Distress Inventory (PDI). However, the main focus of this study was the sensory perception.

The assessments at two time points made it possible to study changes in perceived threat during the casualty chain. The level of perceived threat was moderately but significantly higher at *the scene of the injury *than *in the hospital*, but there was a stronger explained variance measured *in hospital*. The mean score of dissociation and perceptions were quite similar at both measurement points. Measuring the responses *in hospital *seems to be sufficient in identifying those at risk of developing posttraumatic stress.

### Strengths and Limitations

The CCI showed strong internal consistency and a two-factor scale, despite the fact that the participants were drawn from a physically injured population with a broad range of stress symptoms. Accordingly, the instrument can be used in conscious patients admitted in the ER following a physical incident to see who may be at risk for subsequent posttraumatic stress. It examined a large sample from a region surrounding the capital of Norway. The duration of the threat was assessed by questions about *the scene of the injury *and about the participants' stay *in the hospital*.

The participants completed the questionnaires some weeks after their accident. The time of assessment (weeks after the accident) raise questions regarding the CCI's ability to identify patients at risk. Even though a recall bias may be present, those with symptoms after some weeks are most likely at greater risk for symptoms also at a later stage. Analysis showed no significant difference in stress score (IES) between patients answering close to the accident compared to those answering several weeks after the event. This may confirm that the ability to remember feelings and responses in certain situations should not be underestimated. In the pilot study, patients were assessed within a few days post trauma while admitted to hospital. For most patients the self-assessment was difficult at this time point. Some were sleepy, some stressed and some were cognitively not able to concentrate. This was a major reason for postal assessment after discharge. In the same vein, it would have been troublesome for physically traumatized patients in this study to answer questions at the scene of injury and on arrival at the hospital. A number of patients underwent acute surgery. All patients were admitted to an ICU, but some left shortly after admission. In order to make the assessments in a physically stabilized stage of recovery, all patients completed the assessments after discharge.

The CCI measured at two time points was answered at the same time and together with the IES and the PTSS-10. This may have influenced patient's responses by reporting more similar answers at the two measure time points, when analyzing 3 and 12-month data together with baseline data this will no longer represent a problem.

## Conclusion

The CCI measured in hospital appears to be a useful screening instrument for identifying patients at risk for posttraumatic stress symptoms.

## Competing interests

The authors declare that they have no competing of interests.

## Authors' contributions

LaSk, EH and OE conceived and designed the study. LaSk collected the data, and drafted the manuscript. LaSk and LeSa performed the analysis. All authors critically performed interpretation and revision, and approved the final manuscript.

## Pre-publication history

The pre-publication history for this paper can be accessed here:

http://www.biomedcentral.com/1471-227X/11/6/prepub
